# Adaptations in Protein Expression and Regulated Activity of Pyruvate Dehydrogenase Multienzyme Complex in Human Systolic Heart Failure

**DOI:** 10.1155/2019/4532592

**Published:** 2019-02-07

**Authors:** Freya L. Sheeran, Julie Angerosa, Norman Y. Liaw, Michael M. Cheung, Salvatore Pepe

**Affiliations:** ^1^Heart Research, Murdoch Children's Research Institute, Melbourne, Australia; ^2^Department of Surgery at Alfred Hospital, Monash University, Melbourne, Australia; ^3^Department of Paediatrics, University of Melbourne, Royal Children's Hospital, Melbourne, Australia

## Abstract

Pyruvate dehydrogenase (PDH) complex, a multienzyme complex at the nexus of glycolytic and Krebs cycles, provides acetyl-CoA to the Krebs cycle and NADH to complex I thus supporting a critical role in mitochondrial energy production and cellular survival. PDH activity is regulated by pyruvate dehydrogenase phosphatases (PDP1, PDP2), pyruvate dehydrogenase kinases (PDK 1-4), and mitochondrial pyruvate carriers (MPC1, MPC2). As NADH-dependent oxidative phosphorylation is diminished in systolic heart failure, we tested whether the left ventricular myocardium (LV) from end-stage systolic adult heart failure patients (*n* = 26) exhibits altered expression of PDH complex subunits, PDK, MPC, PDP, and PDH complex activity, compared to LV from nonfailing donor hearts (*n* = 21). Compared to nonfailing LV, PDH activity and relative expression levels of E2, E3bp, E1*α*, and E1*β* subunits were greater in LV failure. PDK4, MPC1, and MPC2 expressions were decreased in failing LV, whereas PDP1, PDP2, PDK1, and PDK2 expressions did not differ between nonfailing and failing LV. In order to examine PDK4 further, donor human LV cardiomyocytes were induced in culture to hypertrophy with 0.1 *μ*M angiotensin II and treated with PDK inhibitors (0.2 mM dichloroacetate, or 5 mM pyruvate) or activators (0.6 mM NADH plus 50 *μ*M acetyl CoA). In isolated hypertrophic cardiomyocytes *in vitro*, PDK activators and inhibitors increased and decreased PDK4, respectively. In conclusion, in end-stage failing hearts, greater expression of PDH proteins and decreased expression of PDK4, MPC1, and MPC2 were evident with higher rates of PDH activity. These adaptations support sustained capacity for PDH to facilitate glucose metabolism in the face of other failing bioenergetic pathways.

## 1. Introduction

Heart failure is a syndrome that involves postmyocardial injury adaptations and remodelling at structural, cellular, humoral, and molecular levels that are evoked to maintain viable cardiac output and systemic circulation. However, a feature of progressively deteriorating myocardial systolic function is deficiency in energy supply due to diminished mitochondrial oxidative phosphorylation rates and thus reduced ATP synthesis and creatine phosphate (PCr) stores [1–3]. Relative to nonfailing donor LV, we have previously measured decreased activity rates of mitochondrial respiratory enzyme complex I, complex IV, and nicotinamide nucleotide transhydrogenase; the Krebs cycle enzymes isocitrate dehydrogenase, malate dehydrogenase, and aconitase; plus decreased cellular levels of total glutathione and coenzyme Q_10_, in end-stage failing adult human heart biopsies [4, 5]. In these failing myocardial tissues, diminished management of reactive oxygen metabolism augments postoxidative modifications of key mitochondrial proteins and lipids that inhibit enzyme activity [5], cause loss of membrane cardiolipin [6], decrease the activity of the creatine kinase pathway [7], and impede contractile function of myofibrillar proteins [8].

**Figure 1:**
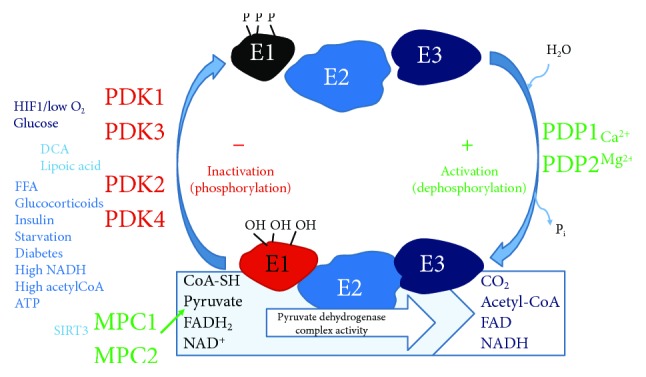
Scheme of pyruvate dehydrogenase complex regulation. Pyruvate dehydrogenase (E1) performs decarboxylation of pyruvate and reductive acetylation of lipoic acid which binds dihydrolipoamide transacetylase (E2). E2 donates protons and electrons to the FAD of dihydrolipoamide dehydrogenase (E3) which converts dihydrolipoic acid and NAD+ into lipoic acid and NADH, reoxidizing E3. Phosphorylation of E1*α* by pyruvate dehydrogenase kinases (PDK1-4) inactivates E1 and subsequently the entire complex. Phosphorylation of the E1*α* subunit is reversed by pyruvate dehydrogenase phosphatase (PDP) and stimulated (via PDK regulation) by insulin, phosphoenolpyruvate, AMP, Ca^2+^, and Mg^2+^ and is competitively inhibited by ATP, NADH, and acetyl-coenzyme A (Acetyl-CoA). MPC1, MPC2 = mitochondrial pyruvate carrier heterodimer; DCA = dichloroacetate; FFA = free fatty acids; SIRT3 = sirtuin 3; FAD = flavin adenine dinucleotide; NAD = nicotinamide adenine dinucleotide; CoA-SH = coenzyme A.

The pyruvate dehydrogenase (PDH) complex is a large multi-subunit enzyme complex within the mitochondrial matrix, consisting of 4 proteins: pyruvate dehydrogenase (E_1_), dihydrolipoamide transacetylase (E_2_), dihydrolipoamide dehydrogenase (E_3_), and one structural protein (E_2_/E_3_-binding protein) [9]. PDH catalyses the irreversible conversion of pyruvate to acetyl CoA, the first intermediate of the Krebs cycle, and NADH required by respiratory complex 1. Thus, PDH is a crucial “gateway” regulator of cellular metabolism, linking the Krebs cycle and subsequently oxidative phosphorylation and ATP synthesis with glycolysis and gluconeogenesis as well as lipid, ketone, and amino acid metabolism [9–15]. Although human PDH relies on mitochondrial pyruvate carrier heterodimer protein function (MPC1, MPC2), PDH activity is tightly controlled by phosphorylation of the E1*α* subunit via PDH kinases (PDK1, PDK2, PDK3, and PDK4) and dephosphorylation by PDH phosphatases (PDP1, PDP2). Thus, PDH regulation occurs at multiple levels, including transcriptional regulation, allosteric regulation, and feedback modulation from metabolic substrate availability, i.e., low oxygen (PDK1), high substrate concentrations such as acetyl CoA/NADH (PDK2), ATP (PDK3), or nutrient deprivation (PDK4). See Figure 1.

As deficits in NADH-dependent mitochondrial complex I activity and oxidative phosphorylation are a feature of human systolic heart failure and most studies of PDH function in heart failure have examined animal models [15], the aims of this study were to measure PDH activity, protein complex subunit expression, and the protein expression of PDH regulatory kinases and phosphatases in left ventricular biopsies from adult human end-stage systolic heart failure and nonfailing donor hearts.

## 2. Materials and Methods

### 2.1. Myocardial Sampling and Processing

Left ventricular myocardial biopsies were obtained from nonfailing (52 ± 3.2 yrs old, left ventricular ejection fraction of 64 ± 3%, *n* = 21) and explanted end-stage failing human hearts (50 ± 2.7 yrs old, NYHA Class IV, left ventricular ejection fraction of 24 ± 2%, *n* = 27). Mid left ventricular endocardial samples (1 g) were snap-frozen upon collection and stored at -80°C. Patient consent was obtained from the Alfred Hospital (Melbourne, Australia) at the time of heart transplantation. Patients with ischemic cardiomyopathy had previously been treated with statins, diuretics, ACE inhibitors, *β*-adrenoreceptor antagonists, and Ca^2+^ antagonists. Nonfailing hearts were obtained following brain death due to subarachnoid haemorrhage in donor patients with no history of overt cardiovascular disease; however, these hearts were excluded from transplantation due to technical complications. The study was approved by the Alfred Hospital Human Ethics Committee for Discarded Tissue Research, and donor heart use was approved for research by donor family consent and the Victorian Organ Donation Service, Australian Red Cross. The myocardium was homogenized under liquid N_2_, and protein estimates were performed as previously described [5].

### 2.2. Myocardial Enzyme Expression and PDH Activity

Protein expression of PDH subunits, PDK1, PDK2, PDP1, PDP2, MPC1, and MPC2 was determined using Western immunoblotting following standard procedures. Briefly, 20 *μ*g protein/lane was loaded onto a 10% SDS-PAGE gel and run at 100 V for 2 hours. Protein bands were transferred to a PVDF membrane at 100 V for 1½ hours and membranes blocked with TBS-Tween (TBST) + 0.5% skim milk powder (blocking buffer) using the SNAP i.d. system (Millipore). Membranes were incubated with a primary antibody overnight at 4°C at the following dilutions: PDK1 (Abcam) 1 : 1000; PDK2 (Abcam) 1 : 1000; PDP1 (Abcam) 1 : 250; PDP2 (Abcam) 1 : 250; MPC1 (Cell Signaling) 1 : 1000; MPC2 (Cell Signaling) 1 : 1000; and PDH western blotting cocktail (Abcam), 6 *μ*g/mL). Porin (VDAC, Abcam), 1 : 5000, was used as a loading control. Membranes were then washed and incubated with a secondary antibody (goat anti-rabbit or anti-mouse/HRP, 1 : 2000 (Bio-Rad)), washed, and briefly incubated with ECL chemiluminescent detection reagent (Perkin Elmer) and exposed to film. Band expression was quantified using ImageJ Software (NIH) and normalized to Complex V *δ* subunit (PDH cocktail) or Porin expression (all other antibodies). Expression of PDK4 was quantified using immunocapture-based ELISA kits (Abcam). PDH enzyme activity was determined spectrophotometrically as described by Pepe et al. [16].

### 2.3. Human Cardiomyocyte Hypertrophy *In Vitro*

Human adult left ventricular cardiomyocytes were purchased from PromoCell (Germany) and cultured according to the recommended conditions in 24-well plates (seeded at 20,000 cells/well) or 96-well white tissue culture plates for plate reader measurements (seeded at 3,000 cells/well). Cells were grown for 72 hr in a myocyte growth medium (MGM; PromoCell) under standard cell culture conditions (37°C, 5% CO_2_) and induced to hypertrophy by a further 24 hr incubation with 0.1 *μ*M angiotensin II (Ang II). Following Ang II treatment, cells were treated for 30 min with PDK inhibitors: 0.2 mM dichloroacetate (DCA) or 5 mM pyruvate; or PDK activators: 0.6 mM NADH plus 50 *μ*M acetyl CoA.

Following treatments, PDK4 measurements were made using the PDK4 ELISA assay on cell extracts (MitoSciences). For analysis of cell size, 2 × 10^4^ cells/well were seeded on coverslips in 12-well culture plates for 72 hours, washed gently with PBS, and fixed for 20 minutes at room temperature with 4% paraformaldehyde in PBS (250 *μ*L/well). Cells were washed twice with 500 *μ*L PBS, then permeabilized and blocked for 45 minutes in 500 *μ*L blocking buffer (2% fetal bovine serum, 2% BSA, 0.1% NP-40 in PBS). Cells were incubated with a primary antibody (sarcomeric *α*-actinin 1 : 100, or slow muscle myosin 1 : 400; Sigma Aldrich), in 250 *μ*L blocking buffer, overnight at 4°C, washed 3 × 5 minutes with PBS, and incubated for 1 hour at room temperature with a secondary antibody (goat *α*-mouse IgG_1_/Alexa Fluor 488 (Invitrogen); 10 *μ*g/mL in blocking buffer). Cells were washed 3 × 5 minutes and incubated for 15 minutes with 3 *μ*M DAPI nucleic acid stain (Thermo Fisher), followed by a further three washes. Coverslips were then carefully removed from the wells, inverted, and fixed onto microscope slides using a mounting medium. Cells were visualized using an AxioVision fluorescence microscope. Cell size was estimated as the total area of pixel fluorescence measured in 50 cells per treatment group, from 3 independent experiments using ImageJ software (NIH).

### 2.4. Statistical Analysis

Statistical comparisons of measures (mean ± standard deviation) between the nonfailing and heart failure groups were performed using GraphPad Prism Version 7. Student's *t*-test with unequal variance or a two-way ANOVA with Bonferroni correction for post hoc comparisons was performed to assess the differences among the groups (*in vitro* cardiomyocyte experiments). A probability value of less than 0.05 was considered significant.

## 3. Results

### 3.1. PDH Activity

**Figure 2:**
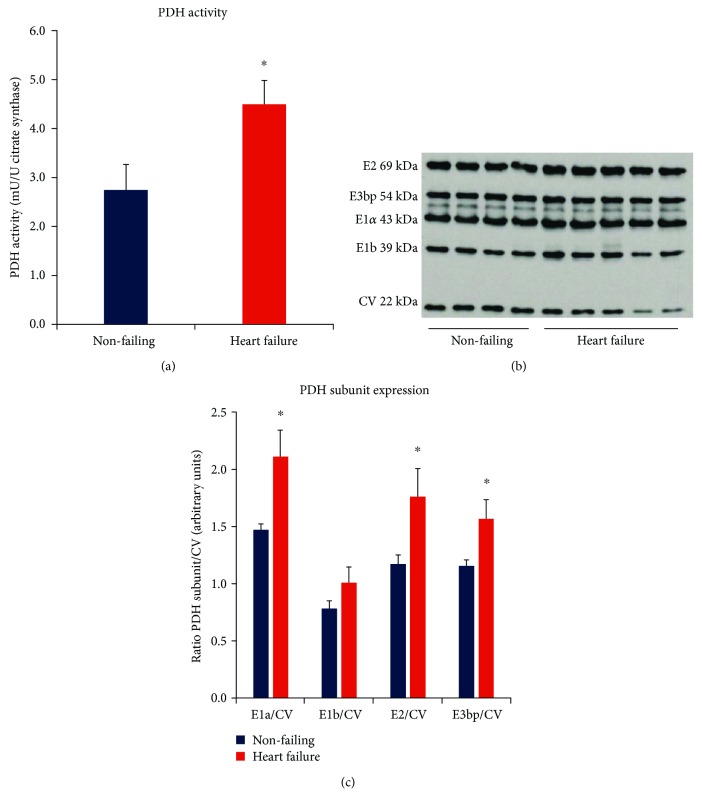
(a) Relative PDH activity in nonfailing (*n* = 21) and heart failure (*n* = 26) groups normalized to citrate synthase (^∗^*p* < 0.05 vs nonfailing). Citrate synthase protein expression did not differ between groups. (b) Example of PDH subunits and inner mitochondrial membrane CV detected concurrently by Western blot. CV = complex V, ATP synthase F_1*α*_ subunit. (c) Mean (±SD) protein expression of PDH subunits E2, E3bp, E1a, and E1b, determined by Western blotting (^∗^*p* < 0.05 vs nonfailing).

PDH activity was significantly increased in the heart failure group when normalized to citrate synthase activity (4.49 ± 0.49 mU/U citrate synthase), representing a 63% increase compared to the nonfailing tissues (2.75 ± 0.51 mU PDH/U citrate synthase, *p* = 0.023) (Figure 2(a)). Comparative activity data from human studies has not previously been reported. However, a rise in PDH activity has been reported in fast-growing cardiomyopathic broiler chickens in the heart failure stage, together with a decline in cardiac function associated with loss of ATP and PCr stores [17]. The amount of active PDH has also been shown to increase in a porcine model of ischemia [18]. However, in a rodent salt-sensitive model of heart failure, no change in PDH activity was seen in either the hypertrophic or failing stages of disease progression [19].

### 3.2. Expression of PDH Enzyme Subunits and Regulatory Enzymes

Figure 2(c) shows the expression profile of PDH enzyme subunits in the heart failure group relative to nonfailing controls. Consistent with an increase in enzyme activity, the expression of all PDH subunits was increased in heart failure (all subunits *p* < 0.05), with the exception of the E1b subunit. The most pronounced increase was for the E1*α* subunit, the regulatory subunit of PDH (*p* = 0.013).

**Figure 3:**
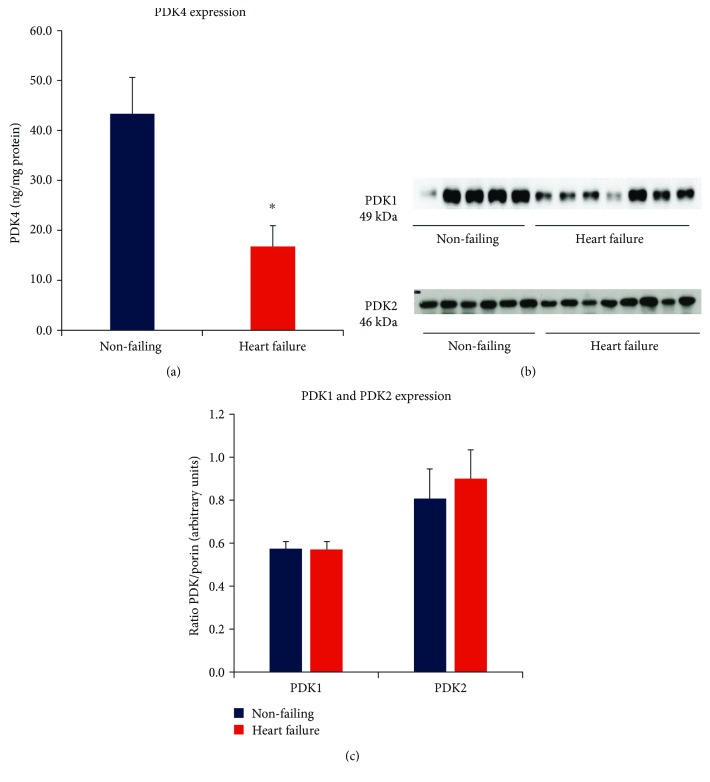
(a) Relative PDK4 levels (ng/mg total myocardial protein) in nonfailing (*n* = 21) and heart failure (*n* = 26) groups measured by ELISA assay (^∗^*p* < 0.05 vs nonfailing). (b) Western immunoblot band examples for PDK1 and PDK2 expression in independent gel assays. Nonfailing and heart failure groups measured concurrently in each gel. (c) Summary of PDK1 and PDK2 protein levels from Western immunoblot assays normalized to porin (VDAC). Myocardial porin protein expression did not differ significantly between groups.

Figure 3(a) summarises PDK4 expression levels which are markedly less in the heart failure group compared to the nonfailing group (*p* = 0.004). As PDK4 is a major cardiac regulatory kinase isoform that reversibly inactivates PDH, decreased PDK4 expression facilitates a greater capacity for activation of PDH in the heart failure group. PDK1 is expressed predominantly in the heart and skeletal tissue and is responsive to low oxygen, whereas PDK2 is activated in response to high availability of NADH and acetyl CoA [12]. The protein expression levels of PDK1 and PDK2 were unaffected by heart failure (Figure 3(c)).

**Figure 4:**
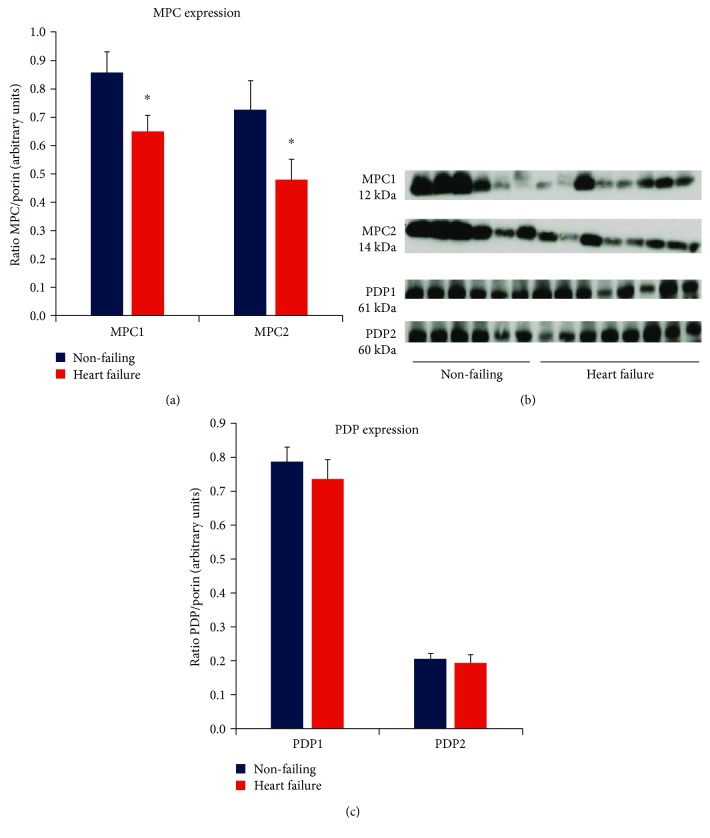
Summary of mitochondrial pyruvate carrier (MPC) and pyruvate dehydrogenase phosphatase (PDP) protein expression determined by Western immunoblot assays. (a) Relative MPC1 and MPC2 protein expression in nonfailing (*n* = 21) and heart failure (*n* = 26), normalized to porin (^∗^*p* < 0.05 vs nonfailing). (b) Western immunoblot band examples from independent MPC1 and MPC2 assays (nonfailing and heart failure groups measured concurrently). (c) Relative PDP1 and PDP2 protein expression (normalized to porin) in nonfailing (*n* = 21) and heart failure (*n* = 26).

Notably, as summarised in Figure 4(a), the failing myocardium expressed significantly less MPC1 and MPC2 compared to nonfailing hearts, indicating reduced capacity for pyruvate uptake into mitochondria and conversion of pyruvate into acetyl-CoA. This is consistent with a previously reported shift towards glycolytic metabolism and lowered PPAR activity in the failing human heart [20, 21]. In contrast, the pyruvate dehydrogenase phosphatases PDP1 and PDP2 did not differ in protein expression level between nonfailing and heart failure groups (Figure 4(c)), indicating that the availability of phosphatases for reversal of E1 phosphorylation is unaffected by severe end-stage heart failure.

### 3.3. PDK4 in Ang II-Dependent Myocyte Hypertrophy *In Vitro*

**Figure 5:**
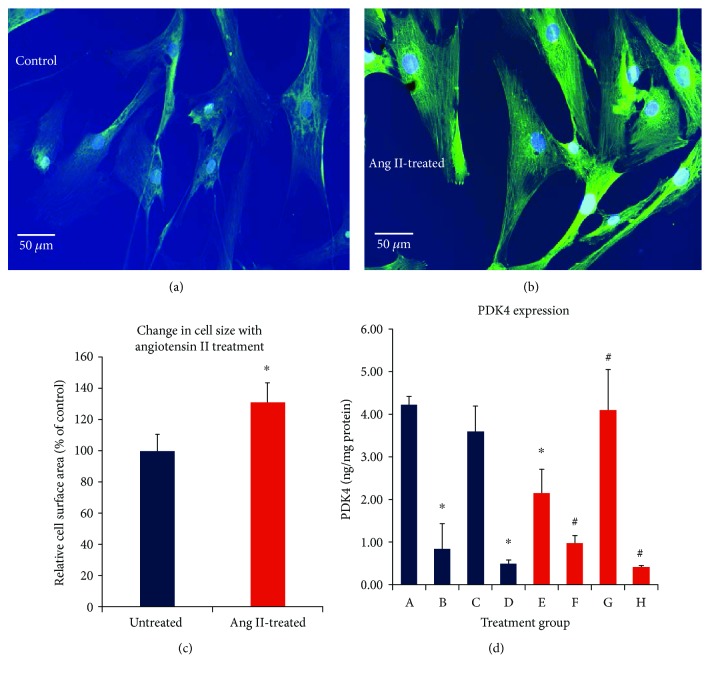
A-H. In vitro culture model of human ventricular cardiomyocytes (groups A-D = control; groups E-H = 0.1 *μ*M Ang II). (a) Control. (b) Ang II-dependent cardiomyocyte hypertrophy. Cardiomyocytes are stained with sarcomeric *α*-actinin (1 : 100) and DAPI (nuclear blue dye). (c) Summary of Ang II-dependent increases in the cell surface area after a 24 hr exposure averaged from 50 cells per treatment group in 3 independent experiments, ^∗^*p* < 0.05 vs Ang II. (d) PDK4 cell protein levels following 30 min treatments with: group A-cell media only; group B-DCA (0.2 mM); group C-NADH (0.6 mM) + acetyl-CoA (50 *μ*M); group D-pyruvate (5 mM); group E-Ang II only (0.1 *μ*M, 24 hrs); group F-Ang II + DCA (0.2 mM); group G-Ang II + NADH (0.6 mM) + acetyl-CoA (50 *μ*M); group H-Ang II + pyruvate (5 mM). ^∗^*p* < 0.05 vs. group A, ^#^*p* < 0.05 vs group E, mean ± SD, *n* = 3 independent experiments.

We tested PDK regulation and expression directly using a simple model to generate cardiac stress and hypertrophy in human ventricular myocytes *in vitro*. In this cell culture model, myocytes were treated for 24 hours with 0.1 *μ*M angiotensin II (Ang II), which has been previously shown to induce hypertrophy and increase production of reactive oxygen species (ROS) in these cells [22] (Figures 5(a) and 5(b)). Cardiomyocyte size increased by 30% following Ang II treatment (Figure 5(c)). Notably, PDK4 expression was lower in Ang II-treated hypertrophic cardiomyocytes than untreated control cells (Figure 5(d)). Exposure for 30 min to the PDK inhibitors DCA (0.2 mM), or pyruvate (5 mM), decreased PDK4 protein expression in both control and Ang II–treated myocytes (groups B and F). Activators of PDK (acetyl CoA and NADH) did not increase PDK4 expression in the control group, as activity may already be at its peak (group C).

However, in the Ang II-treated group, where baseline expression was lower, acetyl CoA + NADH treatment restored PDK4 expression to near baseline control levels (group G). These results demonstrate that Ang II treatment induces adaptive changes in PDK4 expression which can be rapidly modified or reversed by metabolic modulators of PDK activity.

## 4. Discussion

In severe heart failure states, shifts in cardiac metabolism involving decreased oxidative phosphorylation, diminished fatty acid oxidation, and greater reliance on glucose oxidation have been reported predominantly in animal models [15]. In the present study, we examined human myocardial expression and activity of PDH and its key regulatory proteins which to date have not been concurrently examined in detail during end-stage human heart failure. Our main findings include greater PDH activity and E1*α*, E2, and E3bp protein subunit expression levels in the heart failure group, compared to our nonfailing group. Concomitantly, end-stage failing hearts exhibited markedly diminished expression of PDK4, but a mild decrease in MPC1 & MPC2 protein compared to nonfailing donor hearts. In addition, there was no significant difference in E1b, PDP1, PDP2, PDK1, and PDK2 protein levels between the groups. The present findings arising from this “snapshot” of severe end-stage heart failure indicate that the increased PDH activity is contributing to the final stage of adaptive survival in a setting of diminished processes that include decreased activity rates of complex I, complex IV, nicotinamide nucleotide transhydrogenase, isocitrate dehydrogenase, malate dehydrogenase, and aconitase; decreased cellular levels of total glutathione and coenzyme Q_10_; and augmented postoxidative modifications to metabolic and myofilament proteins, as we previously reported for this cohort [4, 5, 8].

Early work with the cardiomyopathic hamster found that decreased PDH activity and decreased total PDH were associated with diminished calcium homeostasis in the failing myocardium [23]. In a rat model of hypertrophic pressure overload, decreased active PDH, without a change in total PDH level, has been reported during the transition from compensated hypertrophy to decompensated heart failure [24]. Although increased PDH activity has been reported in the ischemic porcine heart [18], decreased PDH activity with increased PDK4 has been reported in a porcine model of pacing-induced, early heart failure [25]. In contrast, in early to moderate failure in the microembolized canine heart failure model, PDH activity was unchanged [26], indicating that the shift in metabolism may occur later in heart failure. Kato et al. reported, in a heart failure study of Dahl salt-sensitive rats [19], expression of genes controlling glycolysis, fatty acid oxidation, and mitochondrial function which remained largely unchanged in the hypertrophic phase, decreasing only in the failing stage alongside a decrease in transcriptional regulators. Indeed, in moderate human heart failure (NYHA Class II-III), high rates of fatty acid oxidation and low carbohydrate oxidation relative to healthy subjects have been reported [27], whereas in severe end-stage failure diminished capacity for fatty oxidation and increased glucose metabolism have been reported for NYHA IV heart failure patients [20, 28–30]. Razeghi and colleagues [20] demonstrated that the metabolic profile in heart failure was not due to a reversion to a fetal-like phenotype as previously thought, but by suppression of adult gene transcripts to fetal levels, particularly those involved in fatty acid oxidation and PPARs. The present findings of our study support sustained adaptive capacity for PDH to facilitate glucose metabolism in the face of other failing pathways.

### 4.1. PDH Regulation by PDK and PDP

PDH activity is exquisitely regulated by cellular energy status; i.e., high levels of ATP, NADH, and acetyl-CoA are inhibitory [31, 32]. In addition, there is gene transcriptional regulation of the PDH components, i.e., fasting or metabolic insufficiency downregulates subunit transcription which is restored on feeding or cessation of energetic stress [33]. Rapid control of PDH activation and deactivation is achieved by phosphorylation and dephosphorylation. PDH is tightly regulated by a family of four kinases (PDK1-4), PDK1 and PDK4 being the major isoenzymes in the heart [14], each of which has varying sensitivity to various environmental stimuli and metabolic intermediates. However, a degree of cross-sensitivity occurs between these four PDKs: (a) PDK1 is most involved in sensing low oxygen concentration, (b) PDK2 is sensitive to a high concentration of NADH and a high ratio of acetyl-CoA to CoA ratios, (c) PDK3 is sensitive to a high concentration of ATP, and (d) PDK4 is responsive to nutrient deprivation [14, 34–37]. Three of the four PDKs (PDK 2, 3, and 4) have been reported to be directly under PPAR regulation, thus highlighting their important role in metabolic control [37]. In turn, gene expression of PDK1 is directly activated by HIF1*α* in response to low oxygen levels [38]. Each of the four PDH kinases has varying reactivity on each of the three serine residues of the PDH E1*α* subunit, with phosphorylation of any one of the three serine residues resulting in inhibition of PDH. While each of the PDH kinases is responsive to specific environmental factors, dephosphorylation, and hence activation, of PDH by PDH phosphatase (PDP) is nonspecific, although PDP1 is activated by mitochondrial Ca^2+^ release following muscle contraction, as are the Krebs enzymes, *α*-ketoglutarate dehydrogenase (KGDH), and (NAD)-isocitrate dehydrogenase (ICDH) [11].

In our study, while protein expression of PDK1 and PDK2 was unchanged, PDK4 expression was reduced by more than 60% in the failing heart. Gene expression of PDK1, among other glycolytic genes, is directly activated by HIF1*α* (hypoxia-inducing factor 1*α*) in response to hypoxic oxygen levels, directing metabolism towards glycolysis, maintaining ATP levels, and preventing toxic buildup of ROS [35]. PDK1 expression was unchanged in the heart failure group, suggesting that oxygen delivery is not a significant factor modifying PDH expression in end-stage failure. Nor is nutrient deprivation, as PDK4 expression was low in the heart failure group. PDK2, in comparison, responds to all metabolic markers ATP, NADH, and acetyl CoA and thus serves as one of the major regulators of PDH [14]. Thus, nutrient/substrate availability does not appear to be driving the change in PDH expression. PDK3 expression was not measured due to low expression in the heart [14], whereas mRNA expression changes in PDK2 and PDK4 isoforms have been reported for human heart failure [20]. Our study findings are congruent with studies in PDK4 knockout mice that exhibit increased myocardial PDH activity, increased glucose oxidation, and increased resistance to ischemia-reperfusion injury [39]. Altered PDH subunit expression and PDK regulation may thus provide important adaptive capacity to support mitochondrial bioenergetics in heart failure progression.

### 4.2. Mitochondrial Pyruvate Carrier (MPC)

Pyruvate entry into the mitochondrion through the inner membrane represents a rate-limiting step for pyruvate oxidation and thus oxidative phosphorylation, serving as a critical regulatory point, linking carbohydrate, protein, and fatty acid metabolism. The molecular identity of the pyruvate importer has only been recently characterised as two proteins (MPC1, MPC2) that form a hetero-oligomeric complex in the inner membrane mitochondrial to facilitate pyruvate transport and involves proton cotransport aided by mitochondrial membrane potential whereby an electrically neutral proton gradient drives pyruvate inwards across the inner mitochondrial membrane during proton symport [40]. A surviving myocardium of postischemic porcine heart exhibits increased MPC1 and MPC2 protein levels, similar to myocardial samples from patients with acute ischemia [43].

While the precise mechanisms of regulation remain to be fully elucidated, NAD^+^-dependent sirtuin 3 (SIRT3) has been reported to bind to and deacetylate MPC1 to enhance pyruvate transport activity [44]. SIRT3 deacetylation is also involved in the regulation of other mitochondrial proteins involved in energy production and redox signalling, including enzymes involved with calcium handling and oxidative phosphorylation [45]. It is possible that contributing to the complex maladaptations arising in end-stage heart failure is cumulative lysine acetylation of numerous mitochondrial proteins, including PDH subunits, indicative of a loss of mechanisms involved in deacetylation-dependent regulation, including diminished SIRT3 [46–48]. As measuring protein acetylation status and SIRT deacetylation of mitochondrial proteins in our two groups were beyond the scope of our study, we are not able to determine whether the modest decrease in protein expression of both MPC1 and MPC2 is impacted by a loss of SIRT3 deacetylation in our heart failure group.

### 4.3. Targeting PDK-Dependent Regulation of PDH Activity

DCA is an agent with high specificity for PDK, used orally for over 30 years in the treatment of lactic acidosis in children suffering congenital PDH deficiency and more recently has been proven useful in the treatment of a range of cancers [49]. PDK inhibition with DCA has been shown to provide acute protection against postischemic injury and mechanical dysfunction [18, 34, 50–56]. DCA treatment has been shown to be effective in a Dahl salt-sensitive (DS) rat model of heart failure, by preserving cardiac function and preventing the transition from hypertrophy to failure [19]. Intravenous infusion of DCA (50 mg/kg) for 30 minutes in congestive heart failure patients resulted in improved mechanical efficiency as demonstrated by improvements in cardiac index, stroke work, and stroke volume measures in the time following infusion [53], while improvements in mechanical efficiency and stroke volume have been demonstrated in a select group of patients with chronic angina [54]. Clinical trials in human heart failure patients, however, have not been pursued due to toxicity and management difficulty with chronic DCA use [49].

As we observed a marked decrease in PDK4 expression in our heart failure group, we undertook a preliminary, limited study of whether PDK expression could be modulated in cultured human ventricular cardiomyocytes induced to hypertrophy with Ang II [22]. We found baseline PDK4 expression to be decreased by acute Ang II-treatment in these cardiomyocytes. Treatment with PDK inhibitors, DCA, or pyruvate resulted in a marked inhibition of PDK4 expression in both the control and Ang II groups, demonstrating that expression can be rapidly modified. Expression of PDK4 was not increased with its activators, acetyl-CoA/NADH, in the control group, whereas in the Ang II group, treatment with PDK activators restored expression back up to control baseline levels.

Lon protease is a nuclear encoded, mitochondrial ATP-dependent serine peptidase, which has recently been identified to mediate a vast number of roles in mitochondrial homeostasis including regulation of mitochondrial protein turnover, autophagy, mitochondrial DNA replication, oxidative phosphorylation, mitochondrial morphology, and dynamics [57, 58]. Lon protease is a target of SIRT3 and acts as a chaperone to inner mitochondrial membrane proteins and proteolytic activity for the elimination of damaged proteins and folded regulatory proteins. Lon protease has been shown to specifically degrade cardiac PDK4 [57]. As we did not measure activity or abundance of Lon protease, the role of this enzyme in this context *in vitro* or on the decreased PDK4 seen in our heart failure group is currently unclear, but is an important focus in our ongoing studies on PDK4.

## 5. Conclusions

The results of this study support increased PDH activity in end-stage human heart failure. This increase in PDH activity is facilitated by an increase in PDH protein expression, particularly the E1*α* subunit which forms a key regulatory site of PDH, and a reduction in PDK4 expression which thus limits inactivation of PDH. These key adaptations afford the severely failing left ventricle crucial capacity to utilize glucose-dependent energy production in the face of dwindling energy options. Future work to elucidate mechanisms of altered expression and function for MPC1, MPC2, and PDK isoforms and their adaptations in heart failure progression will be valuable in the setting of mitochondrial energy regulation and the development of clinically viable novel drugs for these targets.

## Data Availability

The data used to support the findings of this study are available from the corresponding author upon request.
